# AR negative triple negative or “quadruple negative” breast cancers in African American women have an enriched basal and immune signature

**DOI:** 10.1371/journal.pone.0196909

**Published:** 2018-06-18

**Authors:** Melissa Davis, Shweta Tripathi, Raymond Hughley, Qinghua He, Sejong Bae, Balasubramanyam Karanam, Rachel Martini, Lisa Newman, Windy Colomb, William Grizzle, Clayton Yates

**Affiliations:** 1 Department of Public Health Sciences, Henry Ford Health Systems and Henry Ford Cancer Institute, Detroit, MI, United States of America; 2 Department of Biology & Center for Cancer Research, Tuskegee University, Tuskegee, AL, United States of America; 3 Department of Chemical Engineering, Auburn University, Auburn, AL, United States of America; 4 Division of Preventive Medicine, University of Alabama at Birmingham School of Medicine, Birmingham, AL, United States of America; 5 Department of Surgery, Henry Ford Health Systems and International Center for the Study of Breast Cancer Subtypes, Detroit, MI, United States of America; 6 Department of Oncology, CHRISTUS St. Patrick Hospital, Lake Charles, LA, United States of America; 7 Department of Pathology, University of Alabama at Birmingham School of Medicine, Birmingham, AL, United States of America; University of South Alabama Mitchell Cancer Institute, UNITED STATES

## Abstract

There is increasing evidence that Androgen Receptor (AR) expression has prognostic usefulness in Triple negative breast cancer (TNBC), where tumors that lack AR expression are considered “Quadruple negative” Breast Cancers (“QNBC”). However, a comprehensive analysis of AR expression within all breast cancer subtypes or stratified by race has not been reported. We assessed AR mRNA expression in 925 tumors from The Cancer Genome Atlas (TCGA), and 136 tumors in 2 confirmation sets. AR protein expression was determined by immunohistochemistry in 197 tumors from a multi-institutional cohort, for a total of 1258 patients analyzed. Cox hazard ratios were used to determine correlations to PAM50 breast cancer subtypes, and TNBC subtypes. Overall, AR-negative patients are diagnosed at a younger age compared to AR-positive patients, with the average age of AA AR-negative patients being, 49. AA breast tumors express AR at lower rates compared to Whites, independent of ER and PR expression (p<0.0001). AR-negative patients have a (66.60; 95% CI, 32–146) odds ratio of being basal-like compared to other PAM50 subtypes, and this is associated with an increased time to progression and decreased overall survival. AA “QNBC” patients predominately demonstrated BL1, BL2 and IM subtypes, with differential expression of E*2F1*, *NFKBIL2*, *CCL2*, *TGFB3*, *CEBPB*, *PDK1*, *IL12RB2*, *IL2RA*, and *SOS1* genes compared to white patients. Immune checkpoint inhibitors PD-1, PD-L1, and CTLA-4 were significantly upregulated in both overall “QNBC” and AA “QNBC” patients as well. Thus, AR could be used as a prognostic marker for breast cancer, particularly in AA “QNBC” patients.

## Introduction

Among women in the United States, breast cancer is the second most common cancer. African-American (AA) women have historically had lower incidence rates relative to White women; however, recent statistics indicate that incidence rates for AA women have converged with those of White women [1]. Relative to White women, AA women are also more likely to be diagnosed at later stages and are 40% more likely to die from breast cancer after the initial diagnosis [1, 2]. Although the underlying cause of this disparity is multifactorial, it is likely that biological factors in the tumors of AA women contribute to the poorer outcomes noted in these patients.

Breast cancer is currently divided into four molecular subtypes by the presence or absence of hormone receptors [(i.e., estrogen (ER) and progesterone (PR)], along with human epidermal growth factor receptor 2 (HER2). This classification influences treatment options and correlates with clinical outcomes such as overall survival (OS) and/or recurrence-free survival [3, 4]. Various reports have suggested that breast tumors lacking AR expression are associated with a shorter disease-free interval and worse OS than those with AR-positive tumors [5–7]. Thus, including AR staining, along with the current standard ER, PR, and HER2 markers has been suggested [5, 8, 9]. This is especially applicable for TNBC patients, since determining AR status would correlate to the sensitivity of these tumors to AR-targeted therapies such as Bicalutamide and Enzalutamide [10, 11]. AR expression, however, is found only in 10%-25% of TNBCs [9–12], and it is associated with favorable survival. Thus, TNBC tumors that lack AR expression are “Quadruple-Negative Breast Cancers (QNBCs)” [6] and could represent a group of patients who have a worse OS and a distinctive biological signature relative to AR-positive TNBCs. Since AA women typically have the most aggressive forms of breast cancer, there is a need to measure the expression of AR in AA patients across all breast cancer subtypes and determine its relationship to clinical outcomes, particularly in TNBC-QNBC patients.

To determine the expression of AR and its relationship to breast cancer subtypes, we compiled a series of Gene Expression Omnibus (GEO) profiles that contained racial and clinical outcomes data totaling 1061 patients. Expression of the AR protein level was confirmed in an additional multi-institutional cohort of 197 breast cancer patients, for a total of 1258 patient evaluated. Relative to White women, AA women had higher percentage (81%) of AR-negative tumors, and, for both races, AR-negative tumors correlated with the basal subtype, a shorter time to progression, and worse OS compared to White women. For AA patients, AR-negative tumors demonstrated a distinctive molecular profile that was enriched for immune genes. 100% of AA TNBC patients were AR-negative. Use of the TNBC subtyping tool showed that, relative to White women, AA women had higher expressions of the basal-like 1 (BL1), basal-like 2 (BL2), and immune modulatory (IM) signatures. These findings suggest that AR is a prognostic marker and should be used routinely along with the standard assessments of ER, PR, and HER2 status to determine tumor aggressiveness, particularly for AA women.

### Statement of translational relevance

We assessed AR expression in a total of 1258 patient at both the mRNA and protein levels, and found that loss of AR is associated with earlier breast cancer diagnosis (3 years earlier in all patients and 7 years earlier in AA patients), a shorter time to progression, and a worst overall survival. We also observed that the absence of AR expression is more prevalent in AA women in all breast cancer subtypes, however AR loss is most frequently observed in TNBC patients, which is referred to as “QNBC”. AA “QNBC” patients have increased basal-like and immune signatures, with the IL12, CCR5, and B-cell response pathways as drivers of this signature. Our data suggest that AR should be added to the current set of ER, PR, and HER2 markers for breast cancer classification, and AA “QNBC” breast cancer patients could be candidates for immune targeted therapies.

## Methods

The Institutional Review Board (IRB) at Tuskegee University and the University of Alabama at Birmingham approved the experimental protocol prior to initiation of the investigation. The BMaP TMA obtained informed consent for all patients used in the generation of TMA from each institution.

### RNA-sequence data set analysis and determination of AR tumor status

With IRB-approved protocols, we determined the RNA-seq output for 925 samples of primary tumors for White or AA women were compiled from the TCGA data portal server website (https://gdc-portal.nci.nih.gov/ accessed on 4/12/2016). AR-status was empirically determined using quantile ranking of AR expression across all samples, and selection of positive vs negative samples was determined by quantile thresholds. Specifically, the subsets of samples below the 25th and above 75th quantiles of the ranked data were determined to be the lowest and highest expression level categories, respectively, corresponding with negative and positive AR status (S1A Fig).

### Microarray data set analysis

GEO files GSE37751 and GSE46581 were downloaded from http://www.ncbi.nlm.nih.gov/geo along with their corresponding platform files. The gene symbol names associated with each read were pulled from each GLP file and merged with its GSE read using the R merge function. Gene expression values for both GSE files were then normalized by the normalization method from the cluster Sim package in R on a scale of <-1, 1> based off positional normalization of the median. The files were then merged using the merge function in R based off their gene symbol name and selected clinical row names.

### Tissue microarray

With IRB-approved protocols for the NCI BMaP initiative, the University of Alabama at Birmingham (UAB), Tulane Medical Center, the University of Mississippi, Emory Medical School, Ponce Medical School, and Moffitt Cancer Center contributed tumors. All tissue microarray (TMA) slides were stained with hematoxylin and eosin (H&E), and stained slides were submitted to the analytical microscopy core for imaging and filing. The biomarker status obtained from Cancer Registry data was confirmed with stains for Her2, PR, and ER using the following antibodies from Ventana Medical Systems: 790–4324 CONFIRM anti-ER (SP1), 790–2223 CONFIRM anti-PR (1E2), 790–100 PATHWAY anti-HER-2/neu (4B5), and (Androgen Receptor antibody (ab133273, abcam, Cambrid13ge, MA). IHC was performed in the Histology Laboratory at the Moffitt Cancer Center using the Ventana Benchmark XT platform following the manufacturer’s specifications.

### Digital scoring of IHC staining

Each core was analyzed individually using the TMA block software associated with Spectrum, then loaded into Tissue Studio v4.0 (Definiens, Munich, Germany). Each core was segmented into tumor and non-tumor components using the Composer functionality for computationally supported histology pattern recognition. This process was manually trained, and quality controlled for accuracy on each core by a pathologist at Moffitt Cancer Center. Within each region (tumor and non-tumor) individual cells were identified using hematoxylin thresholding (0.02), the typical nucleus size was set to be 20 μm^2^, and the cells were grown (cell simulation at 2 μm) in all directions. Image analysis for AR-stained cores was performed using an Aperio Positive Pixel Count® v9. algorithm with the following thresholds: [Hue Value = 0.1; Hue Width = 0.5; Color Saturation Threshold = 0.04; IWP (High) = 220; Iwp (Low) = Ip (High) = 175; Ip(low) = Isp (High) = 100 Isp(Low) = 0] to segment positive staining of various intensities. The algorithm was applied to the entire digital core image to determine the percentage of positive biomarker staining by applicable area and marked as “Percent Expression.” IHC was performed at Tuskegee University and the University of Alabama at Birmingham under IRB-approved protocols.

Samples with values ranging between 0 and 1 were not used. All signature thresholds were set using the Cancer Browser Signatures and Statistics tool. The cutoff range was based on the same principle as the log transformed thresholds, taking the highest and lowest 25–30 percentiles as positive and negative status, respectively. TNBC type data for the TCGA file was obtained by submitting the TNBC patient RNA-seq RSEM values in the illuminahiseq_rnaseqv2-RSEM_genes (MD5) file to http://cbc.mc.vanderbilt.edu/tnbc/. The results were then merged to the clinical data using TCGA IDs.

### Cumulative incidence and Kaplan-Meier curves

For cumulative incidence plots, AR status was determined by its median status and noted as AR-positive or AR-negative. Cumulative incidence plots for time to progression were constructed for all tumors and for basal and non-basal subtype by race. Log-Rank test was used to calculate *P* values. The probabilities of overall survival were calculated using the Kaplan–Meier method and were compared using the log-rank test to calculate *P* values.

### Statistical analyses

The distribution of each clinical variable including age, HR-status AR-status (defined by IHC scores or RNA-seq threshold), race, and stage/grade of the tumor were determined using standard t-tests, ANOVA, and odds ratios with a significance threshold alpha of 0.05. Specific comparisons between continuous variables (i.e., age and AR expression) or categorical variables (i.e., tumor marker status and stage) were measured with a bivariate fit analysis that incorporated a least-squares regression analysis between the two variables.

PAM50 [13, 14] subtype genes were compared with AR tumor expression in the Cancer Genome Browser (https://genome-cancer.ucsc.edu/). TCGA breast carcinoma data were stratified by AR expression levels and dichotomized using a split cut-off based on relative positive or negative (log2 transformed) expression levels.

AR tumor status associations with clinical, demographic variables, and gene expression were determined using pair-wise logistical regression analyses (among continuous variables) or one-way ANOVA/t-tests (among categorical variables). Race and AR-status multiple-testing corrections were applied (Bonferroni) to association p-values with a cutoff of 0.05 to establish significant association. Chi square distributions were used for other categorical variables.

Clustering analyses were used to correlate gene expression trends and discover expression signatures related to AR tumor status. The top 1000 AR-associated genes were analyzed with hierarchical and K-means clustering using R packages obtained from http://cran.fhcrc.org and DANTe Inferno RDN interfaces [15].

## Results

### AR expression in breast cancers differs between AA and White patients and among molecular subtypes

**Fig 1:**
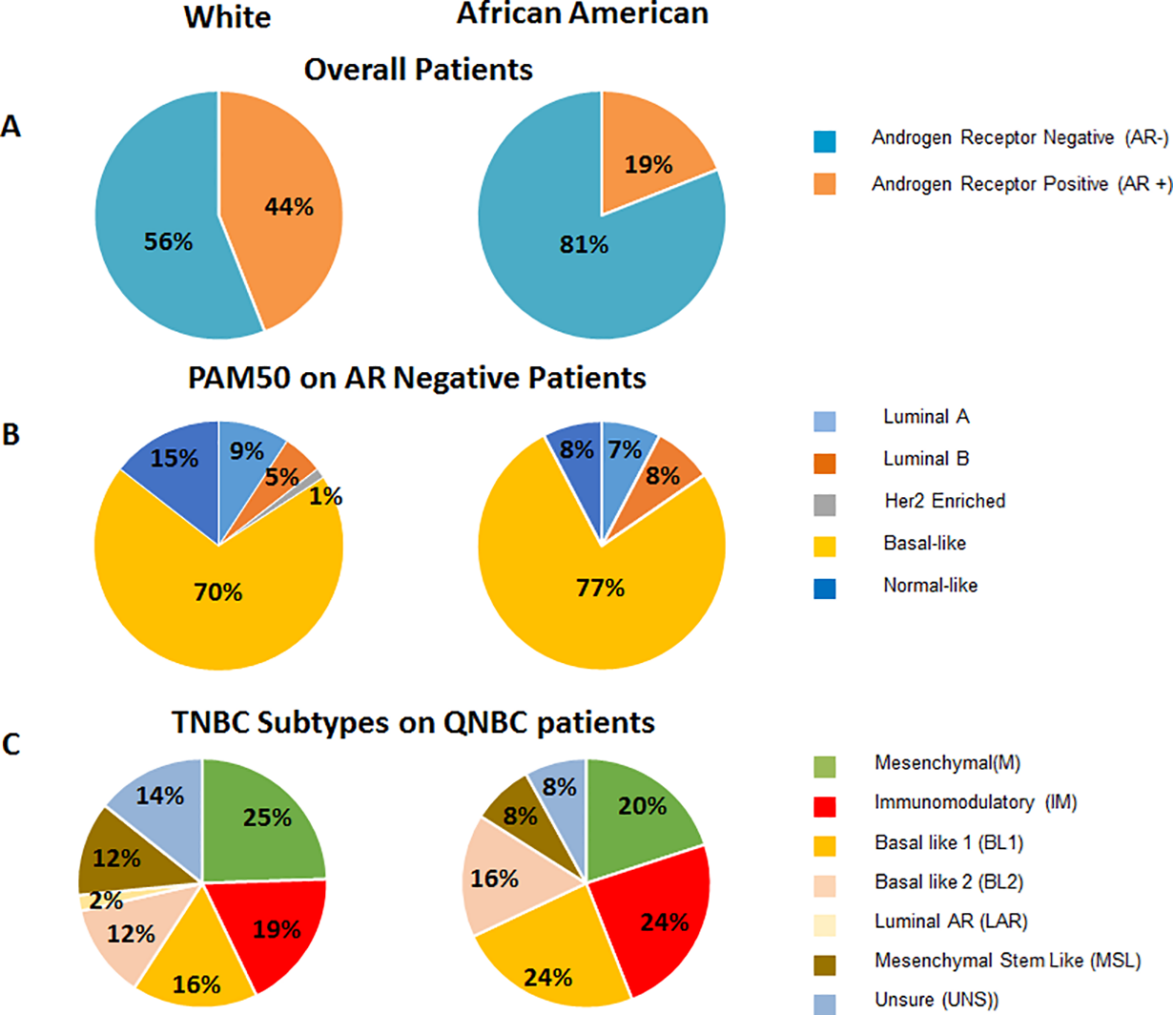
AR status is significantly different between race groups and among molecular subtypes. (A). AA women have more AR-negative tumor types in each molecular subtype. (B). Within the AR-negative subtypes, there are significantly higher proportions of TNBC basal-like. (C). All TNBC samples were subjected to “Vanderbilt” subtypes. AAs, compared to White AR-negative QNBC patients, had more BL1 (24% v 17%), BL2 (16% v 12%), and IM (24% v 19%) subtypes. Inversely, AR-negative White QNBC patients had more mesenchymal (M) (25% v 20%), mesenchymal stem-like (MSL) (12% v 8%), and unstable (UNS) (14% vs 8%) subtypes compared to AR-negative QNBC AA TNBC patients.

Whole-genome expression data from primary breast cancers were obtained from public databases, screened for samples annotated for race information, and analyzed for associations with AR-status among breast cancer subtypes and racial groups (S1A Fig). The results from these analyses were then confirmed in a multi-institutional cohort of breast cancer patients, totaling 1258 patients (S2A Fig). Statistically, there is significant difference between AR-positive and AR-negative patients and the expression of classical breast cancer biomarkers ER, PR, HER2 in the overall population, as well as in African American patients (Table 1); this was even more pronounced in TNBC. We also found that more AA patients (81% v 56%) were AR-negative compared to Whites (Fig 1A). PAM50 analyses showed that majority of AR negative patients are basal-like (Fig 1B). TNBC subtyping of AR-negative (QNBC) patients showed that AA patients had more IM (24% vs 19%) and BL1 (24% vs 16%) compared to Whites (Fig 1C).

**Table 1:** Patient characteristics of TCGA population. The TCGA invasive breast cancer dataset had the largest patient set of RNA-seq data (primary breast cancers for 180 AAs and 745 Whites) was used to quantify distributions of AR expression across patient groups in order to calculate a suitable threshold to stratify the entire dataset/population as AR-positive or AR-negative categories, based on highest and lowest tertiles, exclusively.

**Primary Breast Cancer Characteristics**						
**Variable**	**Analyzable cases (n)**	**AR**				**P value**
		**Positive (n)**		**Negative (n)**		
**Age (Mean)**	925	59		56		
**Total Patients**	925	466		459		
**ER positive**	673	406		267		<0.0001
**PR positive**	583	358		225		<0.0001
**Her2 positive**	116	70		46		0.0439
**TNBC**	**100**	**6**		**94**		**<0.0001**
**Stage1**	172	98		74		0.0551
**Stage 2**	514	247		267		0.1127
**Stage3 and above**	234	118		116		0.9916
**Patient Characteristics Stratified by Race**						
**Variable**	**Analyzable cases (n)**	**AA**		**CA**		**P-value**
** **	** **	**AR+**	**AR-**	**AR+**	**AR-**	** **
**Age (Mean)**	925	56	49	59	53	
**Total Patients**	925	46	134	420	325	
**ER positive**	673	39	68	367	199	0.0142
**PR positive**	583	33	55	325	170	0.0113
**Her2 positive**	116	7	8	63	38	0.2934
**TNBC**	**100**	**0**	**32**	**6**	**62**	**0.0032**
**Stage1**	172	10	22	88	52	0.0011
**Stage 2**	514	26	79	221	188	0.0001
**Stage3 and above**	234	10	33	108	83	0.0001

In the TCGA dataset, we found that AA women with either non-TNBC or TNBC tumor types had lower AR gene expression compared to their White counterparts (S2A–S2C Fig). Additional breast cancer, Gene Expression Omnibus series (GSE) files GSE37751 [16] and GSE46581 [17] that contained annotated race variables as well as HR status showed a significantly lower expression of AR in tumors of AA women compared to those of White women in both non-TNBC and TNBC tumors as well (S2D and S2E Fig). These results indicate that across multiple cohorts, AR expression is lower in tumors from AA women.

AR status was independent of ER-or PR expression status, and AA tumor had highly significant differences in AR status compared to White patients (p<0.001), (S3 Fig). However, within TNBC cases, we observed the most significant race associations with AR-status between AA and White patients (p = 0.0032, with 100% of AA TNBC women displaying AR-negative tumors compared to 91% of TNBC White patients (Table 1).

Similar to TCGA data, IHC analysis of TMA showed that of TNBC patients, AA patients had lower AR protein expression compared to White patients (S1 Table), and this was significant for expression of ER and PR expression, and for TNBC patients. Interestingly, 85% of the TNBC AA patients were AR-negative, compared to only 59% of White patients (S4A Fig). Thus, AR-negative TNBC tumors should be considered QNBC.

### AR expression in breast cancer subtypes correlates with younger age, increased disease progression and lower survival probability for AA women

**Fig 2:**
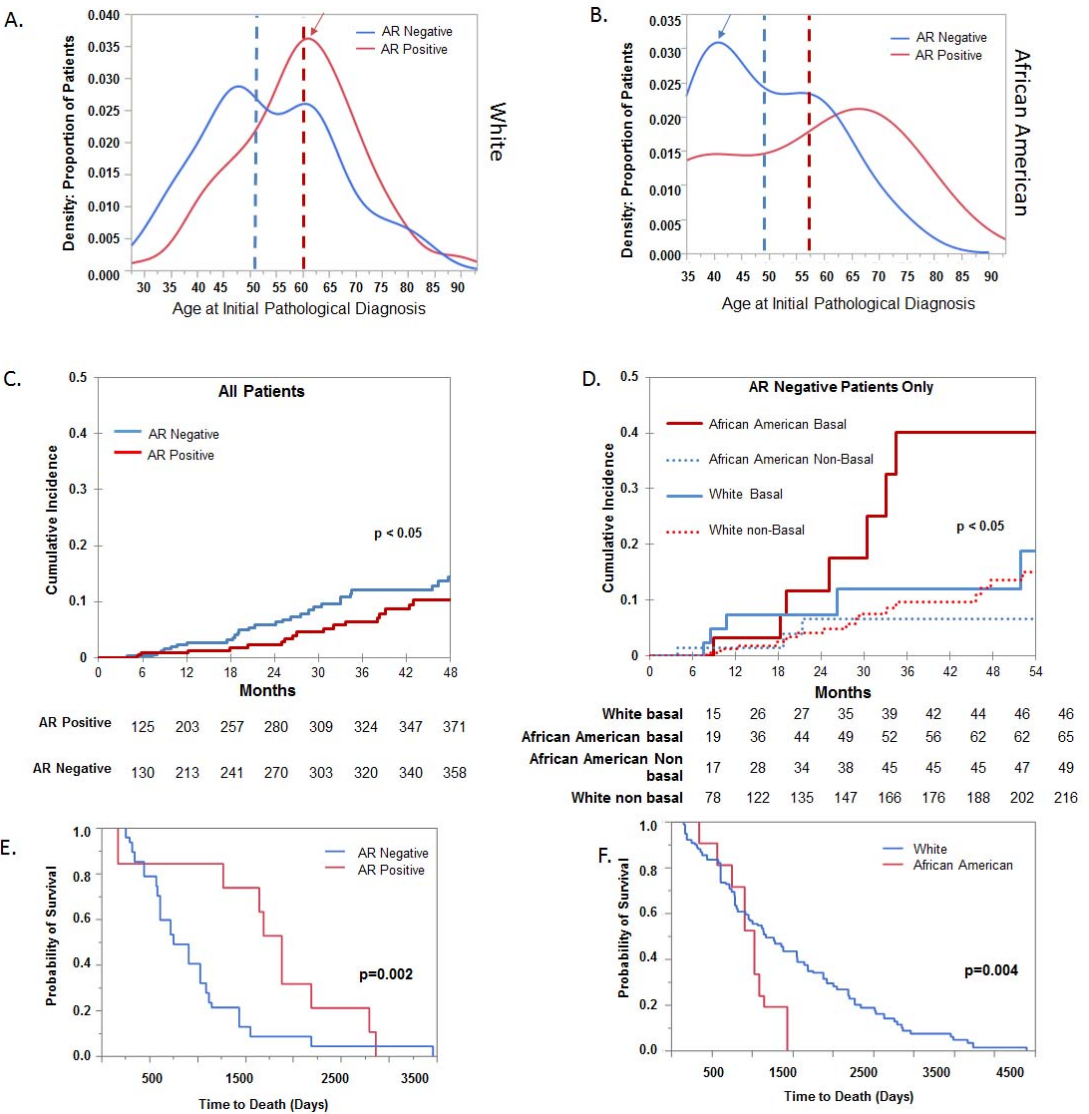
AR tumor status is associated with younger ages and AR-negative patients have a significantly higher rate of disease progression. (A). Density plot of ages for each AR status. The mean age of AR positive subtypes is 59, and the mean age of AR negative subtypes is 56. (B). Sub-stratifying ages by race groups indicates that there is a significant difference in the age for AR negative AA (p = 0.034) as compared to AR positive category. (C). AR negative patients compared AR positive patients have a higher rate of disease progression as determined by cumulative survival analysis. (D) AR negative AA patients with basal subtypes have a higher rate of disease progression, even compared to whites with the same tumor subtype. Log-Rank test was used to calculate *P* values, and significance was determined p<0.05. (E). Kaplan Meier plot shows the overall survival probability in AR-positive and AR-negative patients. (F). Kaplan Meier plot shows the overall survival probability in Whites and African American AR-negative patients.

We observed a significant difference in the age of diagnosis between AR positive vs negative patients with the average age for AR positive patients 59 and AR negative patients had an average age of 56 (p<0.0001) (Table 1). A larger difference is observed when comparing AA and white populations. The average age of diagnosis is 59 for AR positive and 53 for AR negative in whites, while the average age for diagnosis in AA patients is 56 for AR positive and 49 for AR negative in AA (Table 1). This shift in younger age of diagnosis in AA patients is a result of the largest proportion of AR negative patients are within the 35–45 age range (blue arrow) (Fig 2B). However, in whites the largest proportion of patients are AR positive, and are within the 55–65 age range (red arrow) (Fig 2A). This indicates that the shift to younger ages at diagnosis is associated with AR status in the primary tumors.

Cumulative incidence analysis showed that AR-negative patients within all subtypes had shorter times to progression than those for AR-positive patients (p<0.05 regardless of tumor subtype (Fig 2C). Comparing disease progression in both race and tumor subtype revealed that AA patients with AR-negative basal tumors had a much shorter time to progression compared to similar White patients (p<0.05) (Fig 2D).

We also determined that there was a significantly lower overall survival in AR-negative tumors compared to AR positive tumors (Log-Rank p = 0.002). The median time of survival was 1700 days for AR positive tumors and 990 days for AR negative tumors (Fig 2E). Similar results were obtained using kmplot.com that analyzed 1,764 patients, with Log-Rank p = 1.4e-06 (S5 Fig). Since majority of the African American breast tumors where AR negative, we further observed that African American patients showed very early death (1400 days) as compared to their white counterparts (3000 days) (Fig 2F).

### AR negative tumors correlate with the basal and IM TNBC subtypes

Age and Stage adjusted patients with AR-negative tumors (QNBC) had more basal-like tumors than those with AR-positive tumors (TNBC) (70.8% v 3.2%) and a higher odds ratio (OR, 66.60; 95% CI, 32.86–146.06) (Table 2). These patients also had a lower odds ratio with luminal A (OR, 0.073; 95% CI, 0.032–0.144) and HER2-enriched tumors (1.12% vs 8.85%) (OR, 0.17; 95% CI, 0.069–0.002) with a higher probability of expressing the basal-like 1 (BL1) subtype (OR, 4.01; 95% CI, 0.88–38.60) and lower odds of expressing the luminal androgen receptor (LAR) subtype (OR, 0.063; 95%CI, 0.006–0.352) (Table 2).

**Table 2:** PAM50 and Vanderbilt gene expressions subtypes stratified by AR expression in total population (quartile).

Gene Expressions Subtypes Stratified By AR Expression in Total Population					
**PAM50**	**AR-**	**AR+**	**odds ratio**	**95% CI**	**p-value**
**Luminal A**	8	201	0.073	0.032–0.144	<0.0001
**Luminal B**	5	72	0.24	0.086–0.541	<0.001
**Her2**	1	29	0.18	0.07–2303.23	<0.01
**Basal-like**	63	11	66.60	32.86–146.06	<0.0001
**Normal Like**	12	26	1.90	0.902–3.84	0.089
**Vanderbilt TNBC**	**AR-**	**AR+**	**odds ratio**	**95% CI**	**p-value**
**M**	17	3	1.49	0.453–6.22	0.525
**IM**	15	6	0.56	0.187–1.77	0.317
**BL1**	14	1	4.01	0.880–38.60	0.075
**BL2**	10	2	1.43	0.37–8.00	0.623
**LAR**	1	**5**	0.063	0.006–0.352	<0.01
**MSL**	8	4	0.618	0.177–2.39	0.467
**UNS**	9	1	2.16	0.453–21.16	0.363

#### Distinctive gene expression signature in AR-negative tumors of AA and White patients

**Fig 3:**
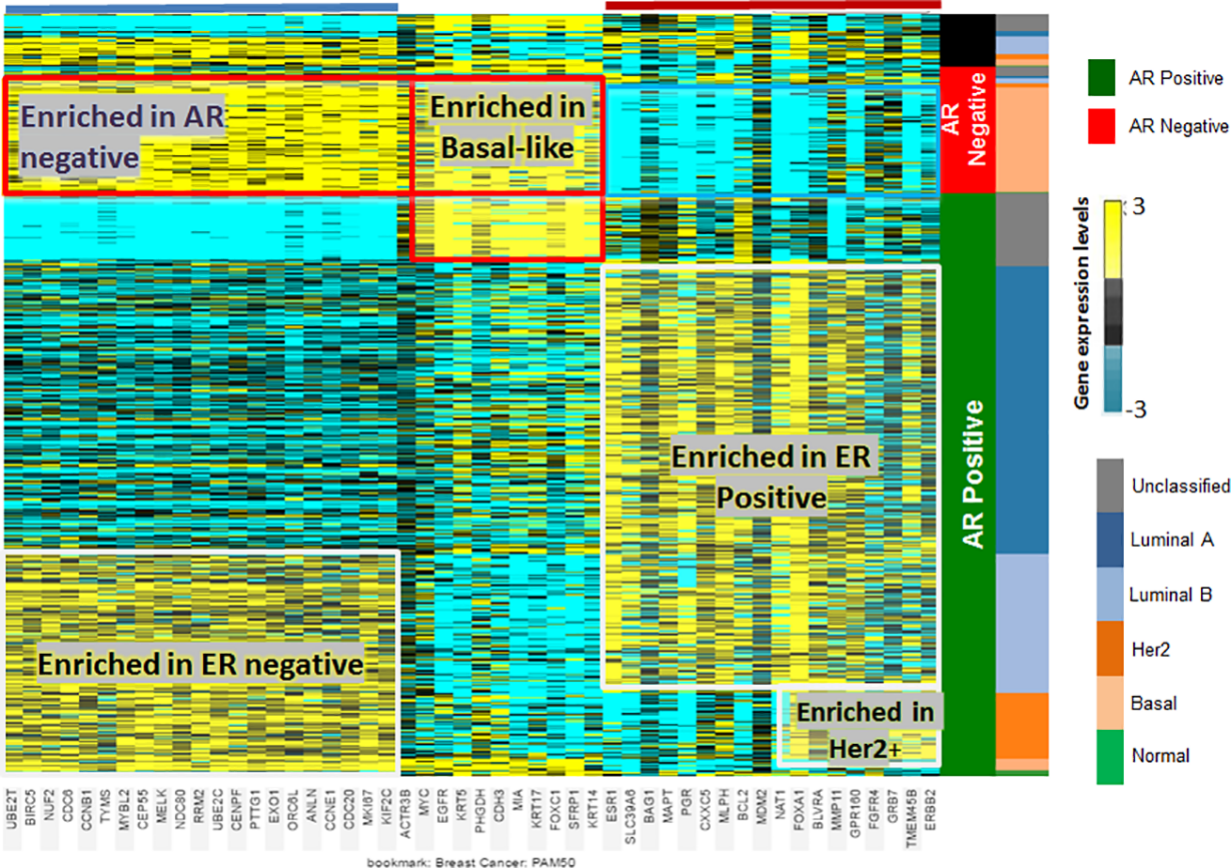
Expression heatmap showing comparison between PAM50 genes subtypes the AR-positive vs AR-negative tumors. **Heat Map using** PAM50 gene signature compared to AR status. Genes that are enriched in ER and AR-negative subtypes (blue bar) show complete absence of expression in a subset of the AR-positive subtypes that is traditionally categorized as ‘unclassified’ subtypes (blue arrow). Genes enriched in the Hormone Receptor (HR) subtypes are typically decreased in the AR-negative subtype (red bar), including some samples that would normally considered ‘unclassified’ or HR-positive.

To determine if the underlying gene signatures of the PAM50 subtypes correlated with AR-status, we compared it to each gene in the PAM50 panel (Fig 3). Each PAM50 subtype displayed a distinct gene signature with regard to AR status. Genes associated with the ER and AR-negative subtypes lack the “unclassified” subtype. Also, genes that are normally enriched in hormone receptor-negative subtypes were also enriched in a subset of AR-positive samples and included genes like *CCNB1* and *BIRC5*. Thus, inclusion of AR status can help classify patients that are not able to be classified by PAM50 designations.

**Fig 4:**
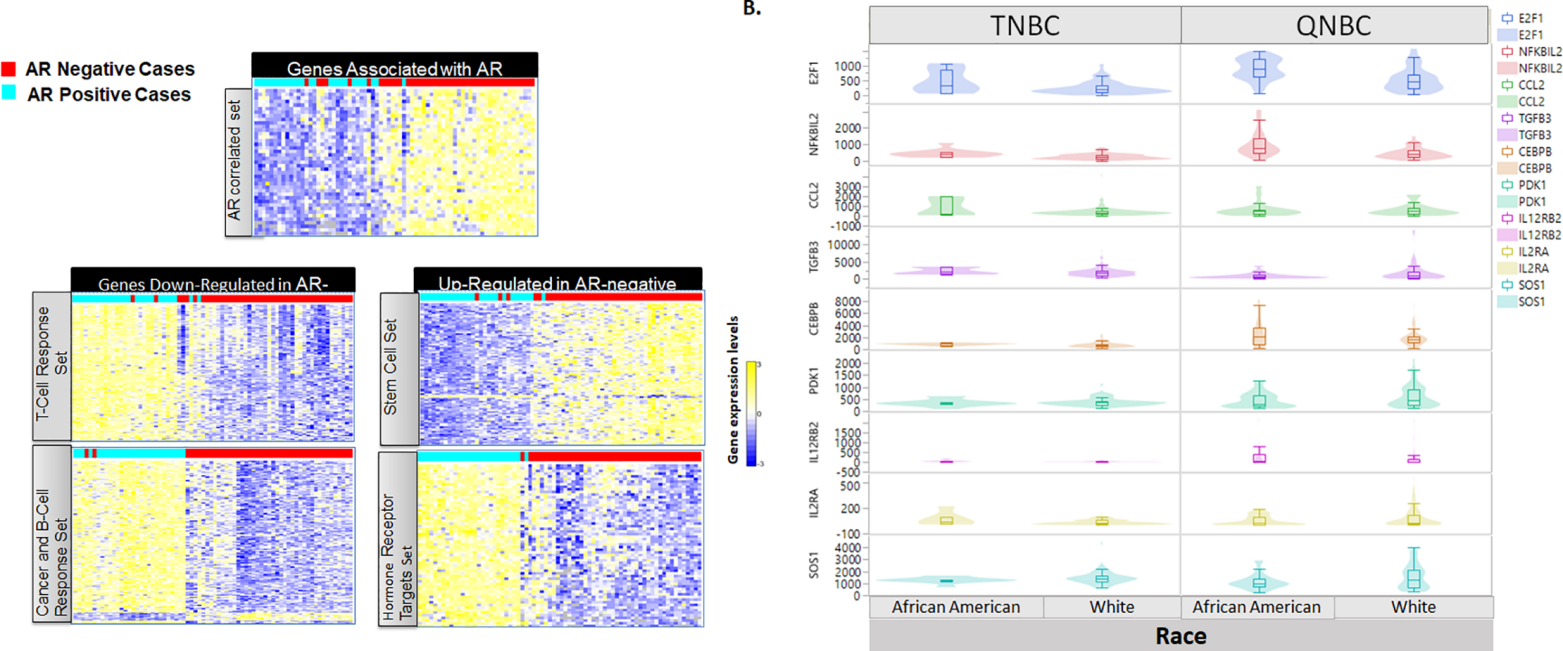
AR-associated genes. A. Genes most highly associated *(bivariate cutoff 1*.*0E-07)* with AR expression across the TCGA dataset were used to determined novel gene expression signatures associated with AR tumor status. Distinct subgroups of genes with shared expression trends were identified using K-means cluster analysis and separated into 5 nodes of genes with expression trends that are either upregulated or downregulated in the AR-negative tumors. B. A subset of genes related to the Immunomodulatory TNBC subtype display statistically significant differences in expression between AA vs White patients when comparing expression in AR-high and AR-low categories.

To determine the specific gene expression profile for AR-negative tumors, we first correlated gene expression trends to AR-status. Significantly associated genes were further analyzed for specific expression patterns using clustering algorithms (Fig 4A). Based on correlated gene expression trends, there were five distinct K-means cluster nodes of AR-associated genes. The node of genes most tightly correlated with AR positive patients was named the “AR-correlated Set,” which is likely genes that are activation targets of AR.

#### Race-specific differences in TNBC-AR associated gene expression

We investigated gene signatures within the AR-negative subtypes that were associated with race. Because AA QNBC patients also show a higher proportion of the IM and BL1 TNBC categories [18], we first determined if genes contained in the subtype pathways also showed an association with AR-status and if this association was race-specific. In both AR-positive and AR-negative subtypes, the genes from IL2RB, CCR5 and B-Cell Antigen receptor pathways showed significant differences (S6A Fig). *CBL*, *CCL2*, *E2F1*, and *NFKBIL2* genes all show a significant difference in expression between race groups (ANOVA p = 0.007, 0.0098, 0.0001, and <0.0001 respectively). *CBL* and *E2F1* showed the most significant expression difference in the AR-negative subtype. Certain groups in general or AR negative group had distinct functional categories. T-cell differentiation had the highest fold enrichment (19.82; p = 9.91E-04), followed by the B-cell receptor signaling pathway (16.95; p = 2.62E-08) and cytokine binding (16.62; p = 2.53E-05) (S2 Table). The cancer and B-cell response set included enrichment of categories related to various cancers as well as enrichment of genes that relate to function/activation of B-cells (S3 Table). This included regulation of immunoglobulin production with highest fold enrichment (13.23, p = 0.02), followed by mammary gland neoplasia (9.16; p = 0.0004) and regulation of production of molecular mediators of immune response (8.39; p = 0.048). The gene set of HR targets included those enriched with specified HR binding sites, with the highest enrichment in vitamin D receptor targets.

Pathway analysis of AR-status and race associated genes revealed an enrichment of immune regulation/response genes, which was also observed comparing TNBC and QNBC patients. These results suggest that a difference in tumor associated immune response depends on AR-status and that both of these mechanisms may be influenced by ethnicity or genetic ancestry. Specifically, the AR and race associated immune-related genes identified in this analysis include; *E2F1*, *NFKBIL2*, *CCL2*, *TGFB3*, *CEBPB*, *PDK1*, *IL12RB2*, *IL2RA*, *and SOS1* (p<0.001) (Fig 4B). Functional pathways related to AR-negative status only include, nuclear mRNA splicing via spliceosome (the most significant GO term, p = 1.27E-05 in upregulated genes), BM CD105+ endothelial expression regulation (p = 2.72E-05 in downregulated genes) (S2 Table). Since the T-cell receptor pathways had the highest fold enrichment, we further probed individual gene expression for CD4 positive and CD8 positive T-cell markers, as well as commonly targeted immune checkpoint genes PD-1, PD-L1, and CTLA-4. Both CD4 (p = 0.01) and CD8B (p = 0.043) have a statistically significant increase in gene expression in QNBC patients compared to non-QNBC patients. However only CD4 (p = 0.04) expression was significantly increased in AA QNBC compared to CA QNBC patients. Interesting all three immune checkpoint inhibitors demonstrate a statistically significant upregulation in QNBC vs non-QNBC patients PD-1 (p = 0.001), PD-L1 (p = 0.037), and CTLA-4 (p<0.0001) (S7A Fig), as well as upregulated in AA QNBC vs CA QNBC PD-1 (p = 0.017), PD-L1 (p = 0.011), and CTLA-4 (p = 0.0114) (S7B Fig)

## Discussion

The addition of AR to the classical triad biomarkers for breast cancers, particularly TNBCs, appears to add a prognostic benefit for clinicians to determine which tumors will be non-aggressive or aggressive [5, 10]. Few reports, however, have examined AR expression in racially diverse populations of patients and correlated expression with related gene signatures. Therefore, we assessed AR gene expression in 925 breast cancers cases within the TCGA dataset. We demonstrated that, compared to White women, AA women had lower expression of AR, as confirmed in two independent datasets. Using mean values as a threshold, 81% of AA women were AR-negative, compared to only 56% of White women. Additionally, loss of AR was independent of other hormone receptors, such as ER and PR, and was associated with an earlier time of breast cancer diagnosis (3 years earlier than AR-positive patients and 6 years earlier for AA patients). AR-negative status was associated with a shorter time to progression, with AR negative AA women demonstrating a shorter time to breast cancer progression and the worst overall survival. Using RNA-based data, within TNBC patients, only 16% express AR, which is similar to other reports [9, 11, 19]. However, after stratifying by race we observed that most AA patients were AR-negative. Similarly, on the protein level, only 6 out of 143 TNBC cases were AR positive in AA patients. Thus, loss of AR in breast cancer patients appears to be a prognostic biomarker, with increased capacity for AA women. This observation provides additional evidence that there is a distinct group of tumors considered as QNBC [6, 20].

AR tumor status is associated with younger ages and there is a clear shift in the age groups between each AR status. The shift indicates that AR-negative status is associated with younger ages. The differences in the mean age of each groups crosses the presumed menopausal status. The median age of AR positive subtypes is 62 (mean = 59), and the median age of AR negative subtypes is 56 (mean = 56). This results in a statistically distinct age range between AR positive vs. negative tumor types. African Americans have a different age distribution among AR-negative cases. Separating the AR status into race, shows a distinct distribution of age between race groups, within AR tumor status categories. There is a significant difference in the age trends for AR negative AA (p = 0.034) as compared to AR positive category but the low case numbers in the AA limit the ability of this analysis to determine if the younger-age trend in AA’s is unique to AR negative status.

Although basal-like tumors account for only about 15% of breast tumors, they are considered as a more aggressive subgroup [21, 22]. Primary tumors from AA women have been typically associated with basal-like characteristics [21, 23, 24]. PAM50 intrinsic molecular subtyping in the TCGA cohort showed that AR-negative tumors had a higher probability of being basal-like in both AA and White women, and there was an inverse probability to be the luminal A subtype. Also, for all patients, AR-negative tumors were strongly associated with the basal-like subtype, with 77% of AR-negative AA women with basal-like tumors compared to 70% of White women. Thus, AR-negative tumors were predominately associated with the more aggressive basal-like subtype.

Most studies examining the expression of AR in breast cancer have concentrated on the TNBC subtype. TNBC, a heterogeneous subtype, has distinct molecular profiles that contribute to clinical outcome and response to therapy [18]. Lehman et al has developed a TNBC subtyping tool which has identified 6 TNBC subtypes. Utilizing this TNBC subtyping tool [18], we found that most of the TNBC tumors we examined are actually QNBCs, which is consistent with previous studies [5, 10, 17, 25], and have a high probability of being associated with the BL1 subtype. Furthermore, 40% of QNBC tumors in AAs are either BL1 (24%) or BL2 (16%) tumors and completely lack the LAR subtype. In contrast, the percentages of tumors with either BL1 or BL2 characteristics were lower for QNBC tumor from White patients, who predominately have the M (25%) or IM (20%) subtype. There was no substantial percentage of AAs with the MSL subtype, a difference between tumors of AA and White patients [26]. Our findings confirm, however, that tumors of AA women have more IM and BL1 characteristics [17, 26]. Although not directly linked, there are reports that AA TNBCs have basal-like tumors and an enriched immune signature [17]. Although there are only a few reports of gene expression differences based on race, our findings are similar to the available data. For example, one report based on use of the TNBC subtyping tool to analyze 69 Caucasian (CA) and 50 AA TNBC patients, found that AA women are more likely to have basal-like subtypes, whereas CA patients are more likely have mesenchymal-like or luminal AR-driven subtypes, which have a more favorable prognosis [27]. They also observed that AA patients have lower AR expression and an enriched immune gene signature [27]. Similarly, investigations of a 699 cohort of TNBCs from Asian women demonstrated that AR-negative tumors represent 62% of TNBCs, and these tumors had an increased likelihood of being basal-like and to recur [25]. These data provide evidence that there is a distinct group of tumors to be considered as QNBC [6, 20].

To determine if AR-negative tumors have a distinctive gene signature, K-means clustering was performed after sorting for PAM50 genes and for AR-positive and -negative status. AR-negative, basal-like tumors had a gene profile distinct from all other subtypes. Analyzing these with gene ontology, we found that, within the basal-like/AR-negative set of patients, AA patients had a distinctive gene signature that could be subdivided into five signatures. Down-regulated genes were enriched in the AR pathway, T cell response, and cancer B cell response. Up-regulated genes occurred in gene sets enriched for stem cells and hormone receptors. There was a difference in immune response and vitamin D signaling. Although NFKB signaling is involved in AA breast cancers [17], two observations were evident from this enriched gene pool. First, there was an association of the AR-negative phenotype with monocyte/macrophage activation. This is consistent with the observation that AA women with breast cancer have more tumor-associated macrophages (TAMs) than White women [28]. Furthermore, this appears to be ancestry-related, as SNPs in *CCL2* and *CCL5*, which are associated with macrophage recruitment to the tumor, were associated with tumors of AA women and increased in AA QNBC patients [29]. Further, in a pilot study of high-grade III, triple-negative tumors of young (mean age 47 years) Nigerian women, there were greater numbers of infiltrating TAMs [30]. There were differences in immune responses associated with vitamin D signaling. For AA men with prostate cancer, vitamin D signaling, and the immune response are linked. In a pilot study of 10 AA men and 14 patients who received vitamin D3 (4000 IU daily) and 13 patients who received placebo for 2 months prior to surgery, the immune response signature was decreased [31]. Most AA patients have a vitamin D deficiency, and our findings of a distinctive immune gene signature in AA QNBC patients suggest a linkage to African ancestry. In further support of this concept, use of the molecular pathways in the TNBC subtyping tool found that E2F1, *NFKBIL2*, *CCL2*, *TGFB3*, *CEBPB*, *PDK1*, *IL12RB2*, *IL2RA*, *SOS1* are differentially expressed in AA QNBC tumors and could act as drivers of this immune response. Of note, for several of these (NFKBIL2, CEBPB, PDK1 and IL12RB2), the trends are in opposite directions in relation to race, indicating that regulation of these genes, in the context of AR status, may be dependent on genetic ancestry/ethnicity. The fact that these genes are molecular drivers within the IL12, CCR5, and B-cell response pathways confirms that, in AA women, immune genes associate with the more aggressive subtypes

Although a clinical threshold that determines AR positivity and negativity for breast cancer patients has not widely established, this is the first report to evaluate the expression of AR, and link the absence of AR to a distinctive gene signature stratified by race. Our results add to the reports suggesting that IHC of AR should be added to the current set of ER, PR, and HER2 markers. More work should be accomplished to determine if the loss of AR is associated with ancestry markers. Immunotherapies to restore T-cell responses, such as PD-1, PD-L1, and CTLA-4, are emerging as anti-tumor therapies for various cancer types, including breast cancer. Although these agents are in ongoing clinical trials for breast [32], our findings that each of these genes are increased in both QNBC and AA QNBC patients provides evidence that QNBC patients could be considered as candidates for this class of therapy. Additionally, several reports have suggested that targeting TAMs or the cytokines that are responsible for the recruitment of macrophages to the tumor microenvironment [33] are also potential targets for cancer therapy. This could represent a potential option for AA QNBC patients with immune signature.

## Supporting information

S1 Fig Distribution of AR expression across breast cancer subtype. A. Comparisons of AR expression between each pair of tumor subtypes. The distribution of AR expression, by ranked quantiles, is shown for each molecular tumor subtype category. This analysis indicates that the TNBC subtypes are composed mainly of the lower quantile cases. There is a shift for Luminal B and Unclassified (typically ER negative) tumor subtypes to contain lower quantile AR-expression cases as well. The table inset indicates the significance (pairwise regression) of differences in AR expression between each subtype. The Student's t ordered differences report for the paired comparisons of AR distribution curves in each subtype (p<0.0001 for each comparison between TNBC vs. Luminal A, Luminal B, unclassified or HER2+; (p<0.001) between Luminal A vs. unclassified; p = 0.038 between HER2+ and unclassified is shown).
(TIF)

S2 Fig Schematic of bioinformatics work flow. A. 1258 patients were assessed in our test and validation sets B. AR Expression stratified by Race in TCGA database C. AR Expression stratified by Race in TCGA TNBC population. D. Overall AR Expression stratified by Race in GSE 37751 database E. AR Expression stratified by Race in TNBC population of GS.
(TIF)

S3 Fig Pie charts showing the correlation of AR expression across all subtypes stratified by race. AR expression was correlated with classical markers ER, PR or HER2. Due to small sample numbers, racial differences in AR expression in the HER2-positive tumor category could not be assessed–of the 46 potential cases, 38 were White women.
(TIF)

S4 Fig Patient characteristics of TMA population. A. IHC staining were digitally scored and box plots show AR protein expression stratified by race in TMA TNBC and non-TNBC patient samples.
(TIF)

S5 Fig Survival analysis of AR positive and AR negative patients from kmplot. A. kmplot.com was used to generate a Kaplan Meier plot shows the overall survival probability in AR-positive and AR-negative patients in 1,764 cases of breast cancer samples across all subtypes. AR Positive vs negative was determined by mean cutoff and Log- Rank test was used to calculate *P* values, and significance was determined (p<0.05).
(TIF)

S6 Fig Race-specific differences in TNBC-AR associated gene expression. A. A subset of genes related to the Immunomodulatory TNBC subtype display statistically significant differences in expression between race groups when comparing expression in AR-high and AR-low categories.
(TIF)

S7 Fig Immune checkpoint gene expression in QNBC patients in TCGA dataset. A. Genes related to immune checkpoint inhibitors were determined in non-QNBC (nQNBC) and QNBC patients. B. Genes related to immune checkpoint inhibitors were determined in AA versus CA QNBC patients. Students Test was used to calculate *P* values, and significance was determined (p<0.05).
(TIF)

S1 Table Clinical characteristics of BMaP breast cancer health disparities TMA. Out of the five GMaP/BMaP regions within the United States, Region 3 consists of Alabama, Georgia, Florida, Mississippi, Louisiana and Puerto Rico. Each institute donated to construct the Breast Cancer TMA. B. Immunohistochemical Staining of AR on TMA slides was performed on tumors from a multi-institutional cohort of 197 patients (74 AA and 123 White) and Chi-Square analysis was performed to determine correlation with clinical pathological features of breast cancer and race.
(DOCX)

S2 Table Gene sets within the IM and BL1 TNBC categories, defined by Lehmann et al, were screened for AR associations. AR and IM associated genes were analyzed for pathway enrichment. The table shows the enrichment of immunological categories. Upregulated functional pathways in relation to AR-negative status. Nodes were collapsed into two over-arching categories to increase statistical power to identify general trends in pathways. Downregulated functional pathways in relation to AR-negative status. Nodes were collapsed into two over-arching categories to increase statistical power to identify general trends in pathways.
(DOCX)

S3 Table Gene ontology of AR associated nodes. Each gene set from the distinct node groups was further analyzed for functional pathway and gene ontology enrichment. Selected functional annotations and Gene Ontologies from each AR status signature node. Certain groups have distinct functional categories. Node 2 includes cancer enrichment and immunological responses. Node 5 includes hormone receptor binding target genes, with the highest enrichment in Vitamin D Receptor targets.
(DOCX)

S4 Table Excel sheet shows the genes associated with the pathways indicating the functional categories embedded in each list. (DOCX)
