# Supplementary figures and images for: AR negative triple negative or “quadruple negative” breast cancers in African American women have an enriched basal and immune signature
Source: PLoS One. 2018 Jun 18;13(6):e0196909. doi: 10.1371/journal.pone.0196909 (PMC6005569; doi:10.1371/journal.pone.0196909)

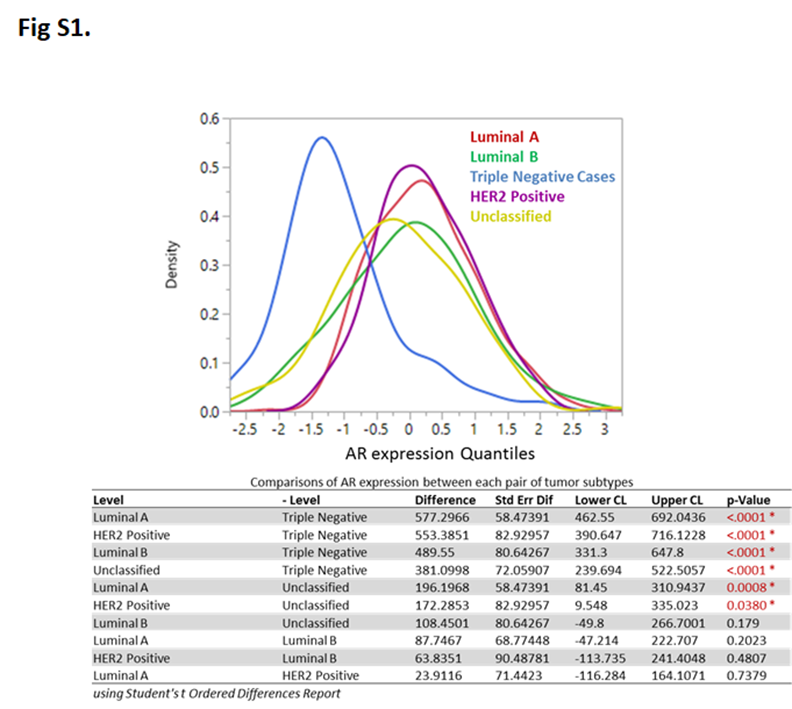

Supplement: S1 Fig — A. Comparisons of AR expression between each pair of tumor subtypes. The distribution of AR expression, by ranked quantiles, is shown for each molecular tumor subtype category. This analysis indicates that the TNBC subtypes are composed mainly of the lower quantile cases. There is a shift for Luminal B and Unclassified (typically ER negative) tumor subtypes to contain lower quantile AR-expression cases as well. The table inset indicates the significance (pairwise regression) of differences in AR expression between each subtype. The Student's t ordered differences report for the paired comparisons of AR distribution curves in each subtype (p<0.0001 for each comparison between TNBC vs. Luminal A, Luminal B, unclassified or HER2+; (p<0.001) between Luminal A vs. unclassified; p = 0.038 between HER2+ and unclassified is shown). (TIF) [file pone.0196909.s001.tif]

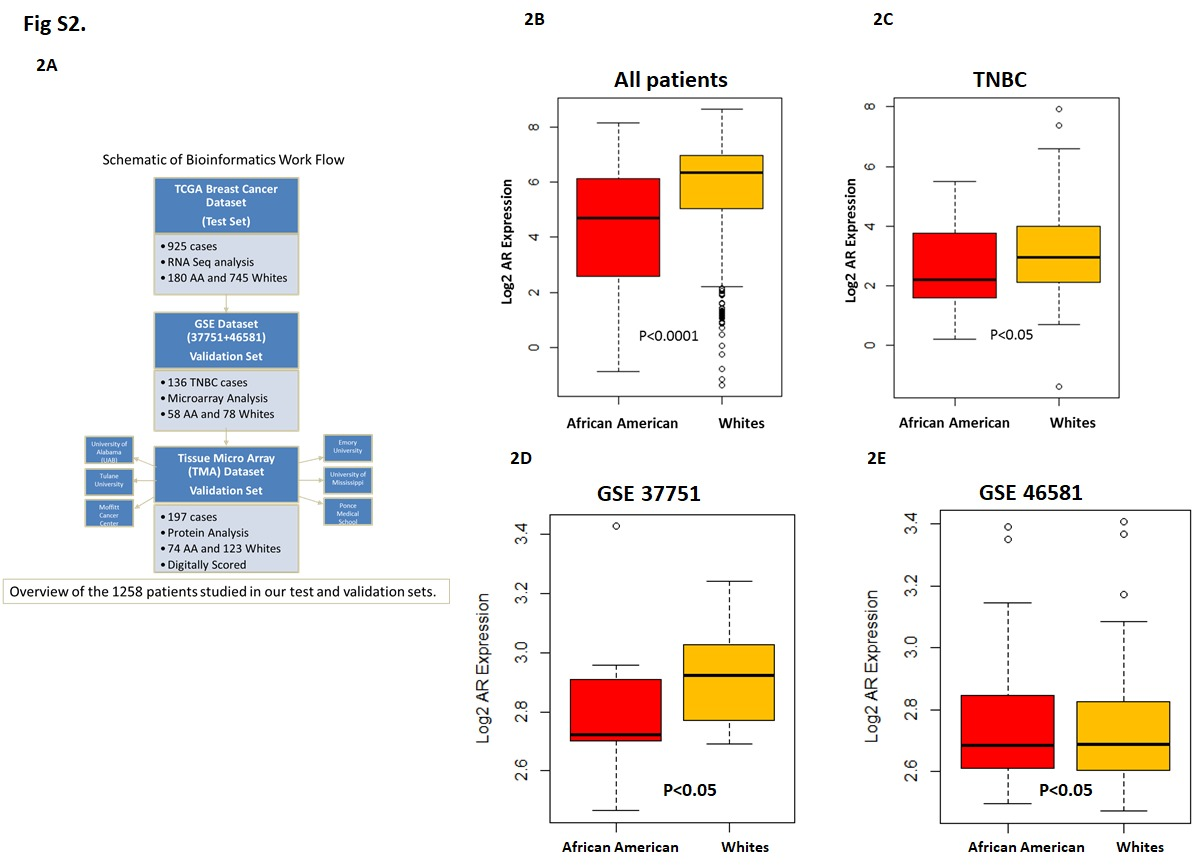

Supplement: S2 Fig — A. 1258 patients were assessed in our test and validation sets B. AR Expression stratified by Race in TCGA database C. AR Expression stratified by Race in TCGA TNBC population. D. Overall AR Expression stratified by Race in GSE 37751 database E. AR Expression stratified by Race in TNBC population of GS. (TIF) [file pone.0196909.s002.tif]

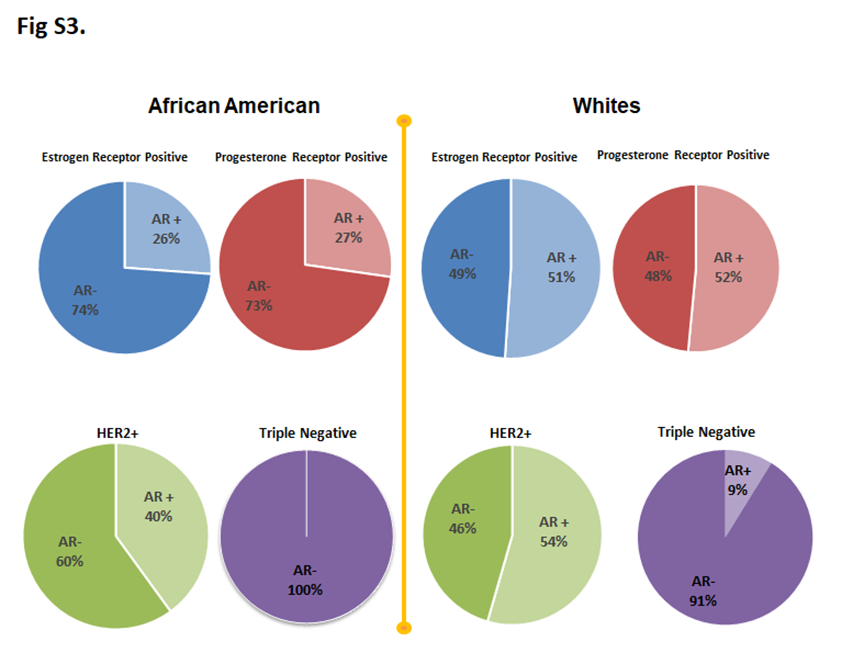

Supplement: S3 Fig — AR expression was correlated with classical markers ER, PR or HER2. Due to small sample numbers, racial differences in AR expression in the HER2-positive tumor category could not be assessed–of the 46 potential cases, 38 were White women. (TIF) [file pone.0196909.s003.tif]

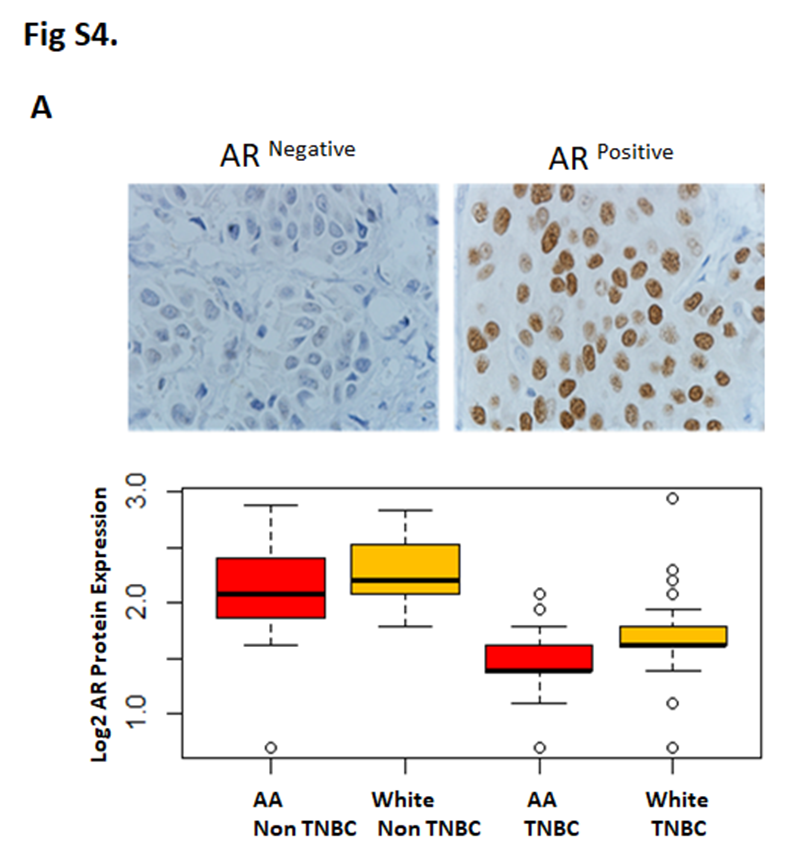

Supplement: S4 Fig — A. IHC staining were digitally scored and box plots show AR protein expression stratified by race in TMA TNBC and non-TNBC patient samples. (TIF) [file pone.0196909.s004.tif]

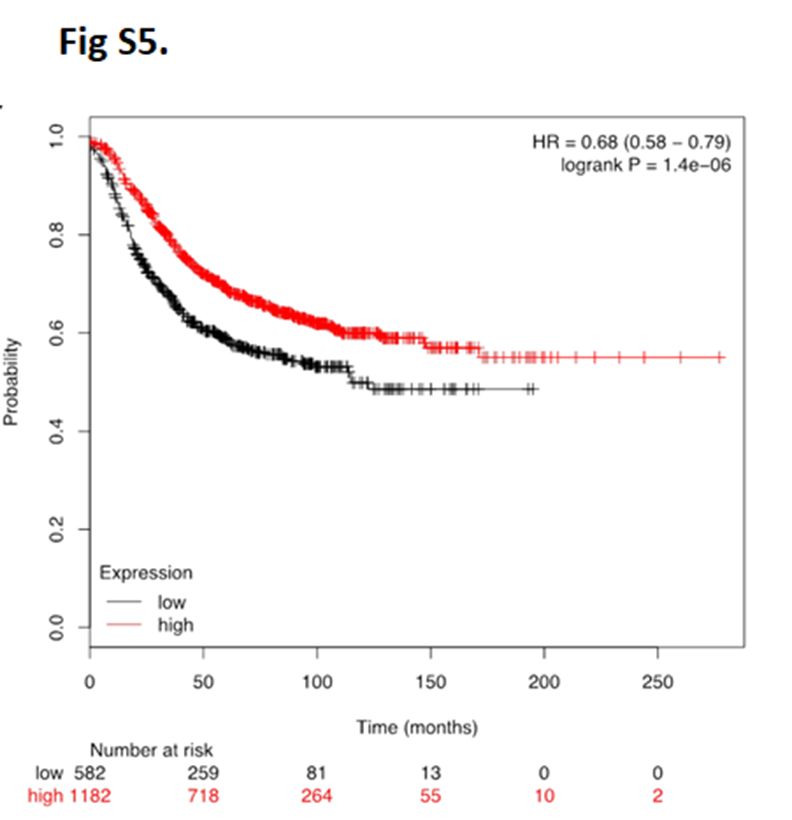

Supplement: S5 Fig — A. kmplot.com was used to generate a Kaplan Meier plot shows the overall survival probability in AR-positive and AR-negative patients in 1,764 cases of breast cancer samples across all subtypes. AR Positive vs negative was determined by mean cutoff and Log- Rank test was used to calculate P values, and significance was determined (p<0.05). (TIF) [file pone.0196909.s005.tif]

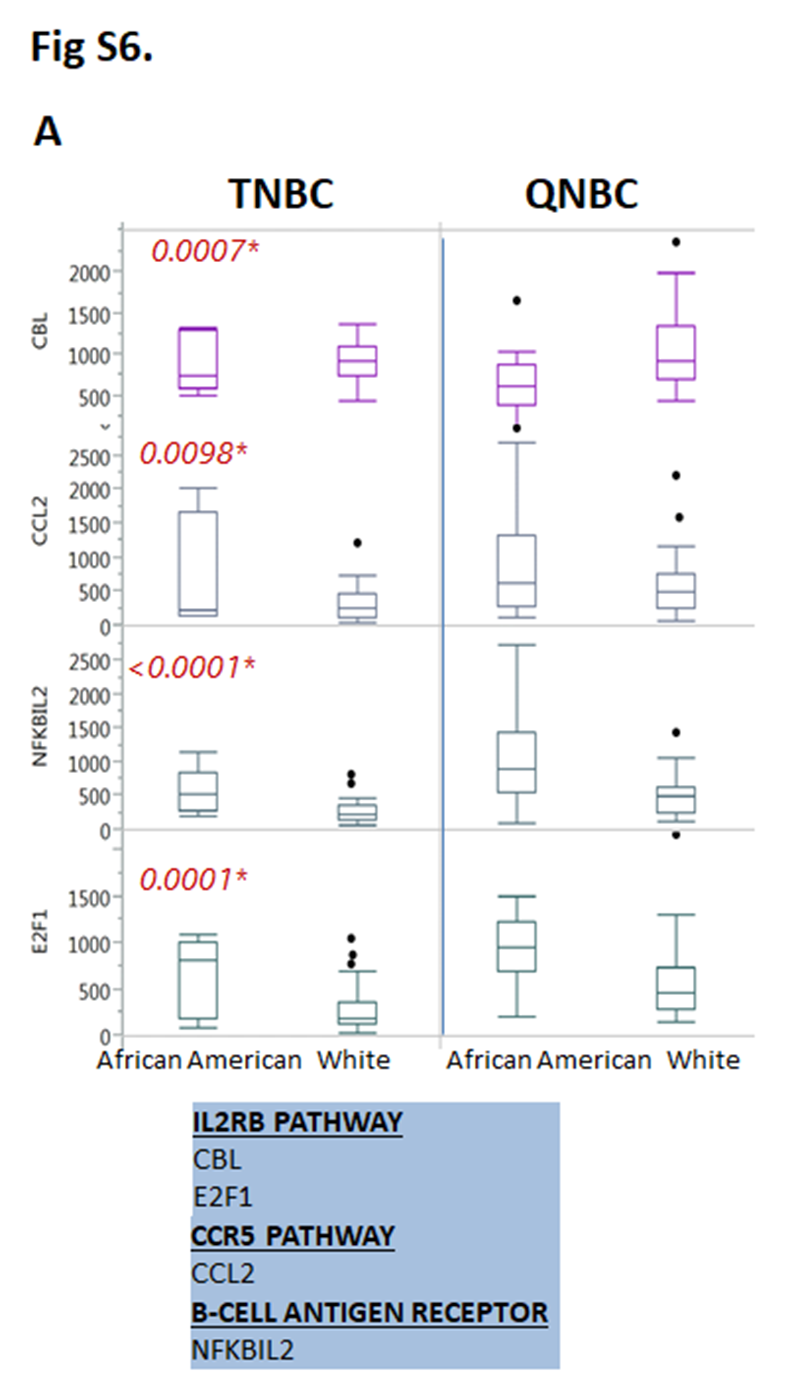

Supplement: S6 Fig — A. A subset of genes related to the Immunomodulatory TNBC subtype display statistically significant differences in expression between race groups when comparing expression in AR-high and AR-low categories. (TIF) [file pone.0196909.s006.tif]

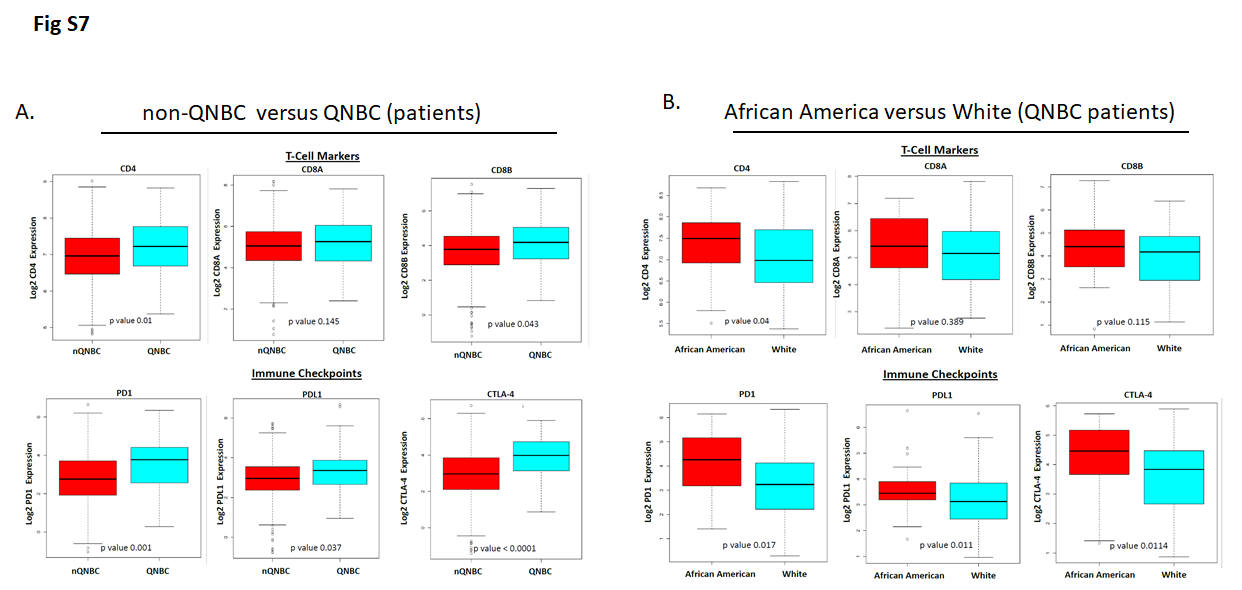

Supplement: S7 Fig — A. Genes related to immune checkpoint inhibitors were determined in non-QNBC (nQNBC) and QNBC patients. B. Genes related to immune checkpoint inhibitors were determined in AA versus CA QNBC patients. Students Test was used to calculate P values, and significance was determined (p<0.05). (TIF) [file pone.0196909.s007.tif]
