# Supplementary material for: AR negative triple negative or “quadruple negative” breast cancers in African American women have an enriched basal and immune signature
Source: PLoS One. 2018 Jun 18;13(6):e0196909. doi: 10.1371/journal.pone.0196909 (PMC6005569; doi:10.1371/journal.pone.0196909)
Supplement: S1 Table — Out of the five GMaP/BMaP regions within the United States, Region 3 consists of Alabama, Georgia, Florida, Mississippi, Louisiana and Puerto Rico. Each institute donated to construct the Breast Cancer TMA. B. Immunohistochemical Staining of AR on TMA slides was performed on tumors from a multi-institutional cohort of 197 patients (74 AA and 123 White) and Chi-Square analysis was performed to determine correlation with clinical pathological features of breast cancer and race. (DOCX) [file pone.0196909.s008.docx]

**S1 Table**

| **Primary Breast Cancer Characteristics (TMA)** | | | | | | |
| --- | --- | --- | --- | --- | --- | --- |
| **Variable** | **Analyzable cases (n)** | **AR** | | | | **p†** |
|  |  | **Positive (n)** | | **Negative (n)** | |  |
| **Age** | 197 | 55 | | 52 | | 0.0674 |
| **Total Patients** | 197 | 100 | | 97 | | 0.8311 |
| **ER +** | 43 | 32 | | 11 | | <0.0001 |
| **PR +** | 33 | 23 | | 10 | | 0.0036 |
| **Her2 +** | 33 | 30 | | 3 | | <0.0001 |
| **TNBC** | **143** | **49** | | **94** | | **<0.0001** |
| **Stage1** | 65 | 35 | | 30 | | 0.5447 |
| **Stage 2** | 83 | 34 | | 49 | | 0.0194 |
| **Stage3 and above** | 38 | 24 | | 14 | | 0.0888 |
| **Stratified by Race** | | | | | | |
| **Variable** | **Analyzable cases (n)** | **AA** | | **CA** | | **p†** |
|  |  | **AR+** | **AR-** | **AR+** | **AR-** |  |
| **Age** | 197 | 53 | 52 | 56 | 53 | 0.0533 |
| **Total Patients** | 197 | 38 | 36 | 62 | 61 | 0.8978 |
| **ER+** | 43 | 23 | 1 | 9 | 10 | 0.0004 |
| **PR+** | 33 | 17 | 1 | 6 | 9 | 0.0015 |
| **Her2+** | 33 | 29 | 3 | 1 | 0 | 1 |
| **TNBC** | **143** | **6** | **33** | **43** | **61** | **0.0036** |
| **Stage1** | 65 | 3 | 3 | 32 | 27 | 1 |
| **Stage 2** | 83 | 20 | 25 | 14 | 24 | 0.4829 |
| **Stage3 and above** | 38 | 15 | 8 | 9 | 6 | 1 |
