# Supplementary material for: AR negative triple negative or “quadruple negative” breast cancers in African American women have an enriched basal and immune signature
Source: PLoS One. 2018 Jun 18;13(6):e0196909. doi: 10.1371/journal.pone.0196909 (PMC6005569; doi:10.1371/journal.pone.0196909)
Supplement: S2 Table — AR and IM associated genes were analyzed for pathway enrichment. The table shows the enrichment of immunological categories. Upregulated functional pathways in relation to AR-negative status. Nodes were collapsed into two over-arching categories to increase statistical power to identify general trends in pathways. Downregulated functional pathways in relation to AR-negative status. Nodes were collapsed into two over-arching categories to increase statistical power to identify general trends in pathways. (DOCX) [file pone.0196909.s009.docx]

**S2 Table**

| **T-Cell Response Set**  **Significant GO Terms** | **P Value** | | | | | **Fold Enrichment** | | | **Benjamini** | | | | | **FDR** | | | | | |  |
| --- | --- | --- | --- | --- | --- | --- | --- | --- | --- | --- | --- | --- | --- | --- | --- | --- | --- | --- | --- | --- |
| **CD4+T-cells_3^rd^-** | 5.87E-11 | | | | | 4.28 | | | *5.7E-13* | | | | | 8.02E-08 | | | | | |  |
| **Cancer and B-Cell Response Set**  **Significant GO Terms** | **P Value** | | | | **Fold Enrichment** | | | | | | | **Benjamini** | | | | | **FDR** | | | |
| **mammary gland invasive breast cancer,**  **ER+, PR+, Her-, grade II** | | 8.24E-08 | | | 3.86 | | | | | | | *7.11E-06* | | | | | 1.12E-04 | | | |
| **alternative splicing** | | 7.13E-06 | | | 1.46 | | | | | | | *7.30E-04* | | | | | 0.009 | | | |
| **white blood cells monocyte** | | 1.37E-06 | | | 2.72 | | | | | | | *7.85E-05* | | | | | 0.002 | | | |
| **mammary gland carcinoma** | 0.0314 | | | | 1.64 | | | | | | | 0.1106 | | | | | 35.333 | | | |
| **PB-CD14+Monocytes** | 0.0058 | | | | 1.16 | | | | | | | *0.0213* | | | | | 5.950 | | | |
| **PB-CD19+Bcells** | 0.0233 | | | | 1.13 | | | | | | | 0.0710 | | | | | 22.102 | | | |
| **Brain benign meningioma of the meningothelial**  **subtype** | 0.0051 | | | | 2.15 | | | | | | | *0.0259* | | | | | 6.720 | | | |
| **mammary gland neoplasia** | 4.84E-04 | | | | 9.16 | | | | | | | 0.0905 | | | | | 0.884 | | | |
| **regulation of immunoglobulin production** | 0.0210 | | | | 13.23 | | | | | | | 0.7410 | | | | | 28.598 | | | |
| **regulation of production of molecular**  **mediator of immune response** | 0.0489 | | | | 8.39 | | | | | | | 0.7473 | | | | | 54.756 | | | |
| **Stem Cell Set**  **Significant GO Terms** | **P Value** | | | | | | | **Fold Enrichment** | | | | **Benjamini** | **FDR** | | | | | |  |  |
| **stem cell expression** | 5.87E-11 | | | | | | | 4.28 | | | | *4.06E-09* | 8.02E-08 | | | | | |  |  |
| **colorectal carcinoma** | 1.02E-10 | | | | | | | 4.80 | | | | *4.41E-09* | 1.39E-07 | | | | | |  |  |
| **nuclear mRNA splicing, via spliceosome** | 7.35E-07 | | | | | | | 11.88 | | | | *8.49E-05* | 0.0011 | | | | | |  |  |
| **white blood cells - monocyte** | 0.0032 | | | | | | | 2.13 | | | | *0.0131* | 4.2761 | | | | | |  |  |
| **AR-Correlated Set**  **Significant GO Terms** | | | **P Value** | | | | **Fold Enrichment** | | | | **Benjamini** | | | | | **FDR** | | |  |  |
| **WHOLE BLOOD** | | | 7.70E-04 | | | | 1.965 | | | | 0.0561 | | | | | 0.8052 | | |  |  |
| **BM-CD105+Endothelial** | | | 0.0021 | | | | 1.968 | | | | *0.0508* | | | | | 2.1641 | | |  |  |
| **Hormone Receptor Targets Set**  **Significant GO Terms** | | | | **P Value** | | **Fold Enrichment** | | | | **Benjamini** | | | | | **FDR** | | |  |  |  |
| **vitamin D receptor binding** | | | | 0.0011 | | 58.39 | | | | *0.046* | | | | | 1.27 | | |  |  |  |
| **hormone receptor binding** | | | | 8.72E-04 | | 20.34 | | | | 0.055 | | | | | 1.01 | | |  |  |  |
| **thyroid hormone receptor binding** | | | | 0.0021 | | 41.97 | | | | 0.067 | | | | | 2.46 | | |  |  |  |
| **nuclear hormone receptor binding** | | | | 5.91E-04 | | 23.25 | | | | 0.073 | | | | | 0.68 | | |  |  |  |
| **PPARA** | | | | 9.53E-04 | | 1.57 | | | | 0.155 | | | | | 1.16 | | |  |  |  |
| **NFKAPPAB65** | | | | 0.0020 | | 2.22 | | | | 0.166 | | | | | 2.49 | | |  |  |  |
| **transcription cofactor activity** | | | | 0.0072 | | 6.16 | | | | 0.172 | | | | | 8.11 | | |  |  |  |
