# Supplementary material for: AR negative triple negative or “quadruple negative” breast cancers in African American women have an enriched basal and immune signature
Source: PLoS One. 2018 Jun 18;13(6):e0196909. doi: 10.1371/journal.pone.0196909 (PMC6005569; doi:10.1371/journal.pone.0196909)
Supplement: S3 Table — Each gene set from the distinct node groups was further analyzed for functional pathway and gene ontology enrichment. Selected functional annotations and Gene Ontologies from each AR status signature node. Certain groups have distinct functional categories. Node 2 includes cancer enrichment and immunological responses. Node 5 includes hormone receptor binding target genes, with the highest enrichment in Vitamin D Receptor targets. (DOCX) [file pone.0196909.s010.docx]

**S3 Table**

|  | | | |  |  |
| --- | --- | --- | --- | --- | --- |
| **Significant GO Terms for Lehmann et. al. subtypes** | | | | | |
| Term | PValue | Fold Enrichment | Benjamini | FDR | |
| B cell receptor signaling pathway | 2.62E-08 | 16.95 | 1.89E-06 | 2.73E-05 | |
| Leukocyte activation | 5.36E-07 | 11.98 | 2.91E-05 | 8.66E-04 | |
| Interleukin 2 | 1.27E-06 | 12.60 | 4.34E-04 | 0.0017 | |
| Defense response | 1.42E-06 | 6.28 | 5.68E-05 | 0.0023 | |
| Response to wounding | 2.93E-06 | 6.68 | 1.00E-04 | 0.0047 | |
| Inflammatory response | 4.87E-06 | 8.92 | 1.49E-04 | 0.0079 | |
| Cytokine binding | 2.53E-05 | 16.62 | 0.0026 | 0.0318 | |
| Lymphocyte activation | 2.75E-05 | 11.33 | 6.45E-04 | 0.0445 | |
| T cell activation | 3.71E-05 | 15.34 | 8.06E-04 | 0.0599 | |
| Immune response | 1.78E-04 | 4.67 | 0.0030 | 0.2878 | |
| T cell differentiation | 9.91E-04 | 19.82 | 0.0120 | 1.5890 | |
| **Significant GO Terms for Upregulated Genes among AR Negative** | | | | |  |
| Term | PValue | Fold Enrichment | Benjamini | FDR | |
| Nuclear mRNA splicing, via Spliceosome | 1.27E-05 | 8.20 | 0.0020 | 0.0195 | |
| Vitamin D receptor binding | 5.16E-04 | 24.81 | 0.1142 | 0.6618 | |
| Thyroid hormone receptor binding | 0.0014 | 17.83 | 0.1499 | 1.7627 | |
| Nuclear hormone receptor binding | 0.0020 | 9.26 | 0.1443 | 2.5264 | |
| Hormone receptor binding | 0.0032 | 8.11 | 0.1191 | 4.0792 | |

|  | | | | |
| --- | --- | --- | --- | --- |
| **Significant GO Terms for Downregulated Genes among AR Negative** | | | | |
| Term | PValue | Fold Enrichment | Benjamini | FDR |
| BM CD105+ Endothelial | 2.72E-05 | 1.42 | 4.25E-04 | 0.0288 |
| Whole Blood | 5.69E-04 | 1.31 | 0.0034 | 0.6007 |
| AF-4 proto-oncoprotein | 0.0022 | 38.67 | 0.4178 | 3.2620 |
