# Supplementary material for: AR negative triple negative or “quadruple negative” breast cancers in African American women have an enriched basal and immune signature
Source: PLoS One. 2018 Jun 18;13(6):e0196909. doi: 10.1371/journal.pone.0196909 (PMC6005569; doi:10.1371/journal.pone.0196909)
Supplement: S4 Table — (DOCX) [file pone.0196909.s011.docx]

**S4 Table**

| **OFFICIAL GENE SYMBOL** | **Name** | **OFFICIAL GENE SYMBOL** | **Name** | **OFFICIAL GENE SYMBOL** | **Name** | **OFFICIAL GENE SYMBOL** | **Name** |
| --- | --- | --- | --- | --- | --- | --- | --- |
| FKRP | fukutin related protein(FKRP) | MAP2K2 | mitogen-activated protein kinase kinase 2(MAP2K2) | AIP | aryl hydrocarbon receptor interacting protein(AIP) | ARHGDIA | Rho GDP dissociation inhibitor alpha(ARHGDIA) |
| UBAP2 | ubiquitin associated protein 2(UBAP2) | PPIG | peptidylprolyl isomerase G(PPIG) | SUV39H1 | suppressor of variegation 3-9 homolog 1(SUV39H1) | CTBP1 | C-terminal binding protein 1(CTBP1) |
| EIF4A3 | eukaryotic translation initiation factor 4A3(EIF4A3) | RABGAP1L | RAB GTPase activating protein 1 like(RABGAP1L) | GSTP1 | glutathione S-transferase pi 1(GSTP1) | RHOA | ras homolog family member A(RHOA) |
| KIF2C | kinesin family member 2C(KIF2C) | TCF7 | transcription factor 7 (T-cell specific, HMG-box)(TCF7) | CHEK2 | checkpoint kinase 2(CHEK2) | DRAP1 | DR1 associated protein 1(DRAP1) |
| ATR | ATR serine/threonine kinase(ATR) | BUB1B | BUB1 mitotic checkpoint serine/threonine kinase B(BUB1B) | ABI1 | abl interactor 1(ABI1) | RSPO4 | R-spondin 4(RSPO4) |
| EXOSC5 | exosome component 5(EXOSC5) | ATXN2L | ataxin 2 like(ATXN2L) | IL4R | interleukin 4 receptor(IL4R) | TIFA | TRAF interacting protein with forkhead associated domain(TIFA) |
| RELB | RELB proto-oncogene, NF-kB subunit(RELB) | MAPK11 | mitogen-activated protein kinase 11(MAPK11) | APAF1 | apoptotic peptidase activating factor 1(APAF1) | SP3 | Sp3 transcription factor(SP3) |
| NRAS | neuroblastoma RAS viral oncogene homolog(NRAS) | ITGB7 | integrin subunit beta 7(ITGB7) | NUP62 | nucleoporin 62(NUP62) | TMTC4 | transmembrane and tetratricopeptide repeat containing 4(TMTC4) |
| NOLC1 | nucleolar and coiled-body phosphoprotein 1(NOLC1) | RUVBL2 | RuvB like AAA ATPase 2(RUVBL2) | E2F2 | E2F transcription factor 2(E2F2) | PML | promyelocytic leukemia(PML) |
| MSC | musculin(MSC) | GMNN | geminin, DNA replication inhibitor(GMNN) | MAP3K2 | mitogen-activated protein kinase kinase kinase 2(MAP3K2) | FAM175B | family with sequence similarity 175 member B(FAM175B) |
| GIMAP5 | GTPase, IMAP family member 5(GIMAP5) | SLC9A3R1 | SLC9A3 regulator 1(SLC9A3R1) | PPP1R12A | protein phosphatase 1 regulatory subunit 12A(PPP1R12A) | CD8B | CD8b molecule(CD8B) |
| HRAS | HRas proto-oncogene, GTPase(HRAS) | HLX | H2.0 like homeobox(HLX) | CASP8AP2 | caspase 8 associated protein 2(CASP8AP2) | NFIL3 | nuclear factor, interleukin 3 regulated(NFIL3) |
| abl1 | ABL proto-oncogene 1, non-receptor tyrosine kinase(ABL1) | MAP2K7 | mitogen-activated protein kinase kinase 7(MAP2K7) | SORT1 | sortilin 1(SORT1) | BCAP31 | B-cell receptor-associated protein 31(BCAP31) |
| ZNF598 | zinc finger protein 598(ZNF598) | SELP | selectin P(SELP) | PBRM1 | polybromo 1(PBRM1) | CRLF3 | cytokine receptor like factor 3(CRLF3) |
| YWHAQ | tyrosine 3-monooxygenase/tryptophan 5-monooxygenase activation protein theta(YWHAQ) | ESR1 | estrogen receptor 1(ESR1) | DUT | deoxyuridine triphosphatase(DUT) | EMD | emerin(EMD) |
| SNAP23 | synaptosome associated protein 23(SNAP23) | PRKDC | protein kinase, DNA-activated, catalytic polypeptide(PRKDC) | LBH | limb bud and heart development(LBH) | CD247 | CD247 molecule(CD247) |
| PARP9 | poly(ADP-ribose) polymerase family member 9(PARP9) | PRKCQ | protein kinase C theta(PRKCQ) | DUSP2 | dual specificity phosphatase 2(DUSP2) | NFATC3 | nuclear factor of activated T-cells 3(NFATC3) |
| HN1 | hematological and neurological expressed 1(HN1) | PKN2 | protein kinase N2(PKN2) | NBN | nibrin(NBN) | MDC1 | mediator of DNA damage checkpoint 1(MDC1) |
| SEC23B | Sec23 homolog B, coat complex II component(SEC23B) | SELPLG | selectin P ligand(SELPLG) | BRMS1L | breast cancer metastasis-suppressor 1-like(BRMS1L) | NFATC1 | nuclear factor of activated T-cells 1(NFATC1) |
| CDCA4 | cell division cycle associated 4(CDCA4) | HNRNPU | heterogeneous nuclear ribonucleoprotein U(HNRNPU) | APOE | apolipoprotein E(APOE) | SRC | SRC proto-oncogene, non-receptor tyrosine kinase(SRC) |
| DBN1 | drebrin 1(DBN1) | SKA3 | spindle and kinetochore associated complex subunit 3(SKA3) | TOR2A | torsin family 2 member A(TOR2A) | rbm12 | RNA binding motif protein 12(RBM12) |
| ING4 | inhibitor of growth family member 4(ING4) | BLM | Bloom syndrome RecQ like helicase(BLM) | CXCL12 | C-X-C motif chemokine ligand 12(CXCL12) | SH2B2 | SH2B adaptor protein 2(SH2B2) |
| ABTB1 | ankyrin repeat and BTB domain containing 1(ABTB1) | ISG20 | interferon stimulated exonuclease gene 20(ISG20) | ME2 | malic enzyme 2(ME2) | NFKBIE | NFKB inhibitor epsilon(NFKBIE) |
| DAXX | death domain associated protein(DAXX) | XRCC2 | X-ray repair cross complementing 2(XRCC2) | LRCH3 | leucine rich repeats and calponin homology domain containing 3(LRCH3) | NFKBIB | NFKB inhibitor beta(NFKBIB) |
| CDC45 | cell division cycle 45(CDC45) | IRF2 | interferon regulatory factor 2(IRF2) | APC | APC, WNT signaling pathway regulator(APC) | EBAG9 | estrogen receptor binding site associated, antigen, 9(EBAG9) |
| PLEKHF2 | pleckstrin homology and FYVE domain containing 2(PLEKHF2) | PLD4 | phospholipase D family member 4(PLD4) | LIMD1 | LIM domains containing 1(LIMD1) | NFKBIA | NFKB inhibitor alpha(NFKBIA) |
| USP24 | ubiquitin specific peptidase 24(USP24) | FAHD2A | fumarylacetoacetate hydrolase domain containing 2A(FAHD2A) | CBLB | Cbl proto-oncogene B(CBLB) | NFKB2 | nuclear factor kappa B subunit 2(NFKB2) |
| DISC1 | disrupted in schizophrenia 1(DISC1) | rnaseh2c | ribonuclease H2 subunit C(RNASEH2C) | PIKFYVE | phosphoinositide kinase, FYVE-type zinc finger containing(PIKFYVE) | NFKB1 | nuclear factor kappa B subunit 1(NFKB1) |
| NOD1 | nucleotide binding oligomerization domain containing 1(NOD1) | PRKCD | protein kinase C delta(PRKCD) | IGBP1 | immunoglobulin (CD79A) binding protein 1(IGBP1) | NDFIP2 | Nedd4 family interacting protein 2(NDFIP2) |
| ZNF43 | zinc finger protein 43(ZNF43) | XPA | XPA, DNA damage recognition and repair factor(XPA) | PDPK1 | 3-phosphoinositide dependent protein kinase 1(PDPK1) | SLC28A1 | solute carrier family 28 member 1(SLC28A1) |
| SNRNP70 | small nuclear ribonucleoprotein U1 subunit 70(SNRNP70) | IRAK1 | interleukin 1 receptor associated kinase 1(IRAK1) | rab11fip5 | RAB11 family interacting protein 5(RAB11FIP5) | RAD23B | RAD23 homolog B, nucleotide excision repair protein(RAD23B) |
| SORL1 | sortilin related receptor 1(SORL1) | SQSTM1 | sequestosome 1(SQSTM1) | BIRC5 | baculoviral IAP repeat containing 5(BIRC5) | EXO1 | exonuclease 1(EXO1) |
| GNL1 | G protein nucleolar 1 (putative)(GNL1) | sec24a | SEC24 homolog A, COPII coat complex component(SEC24A) | TSC22D3 | TSC22 domain family member 3(TSC22D3) | FBXO44 | F-box protein 44(FBXO44) |
| SOS2 | SOS Ras/Rho guanine nucleotide exchange factor 2(SOS2) | TFEB | transcription factor EB(TFEB) | LPP | LIM domain containing preferred translocation partner in lipoma(LPP) | RAD21 | RAD21 cohesin complex component(RAD21) |
| CD83 | CD83 molecule(CD83) | RAB30 | RAB30, member RAS oncogene family(RAB30) | coro1a | coronin 1A(CORO1A) | STMN1 | stathmin 1(STMN1) |
| SOS1 | SOS Ras/Rac guanine nucleotide exchange factor 1(SOS1) | DBNL | drebrin like(DBNL) | RTN4IP1 | reticulon 4 interacting protein 1(RTN4IP1) | RPL28 | ribosomal protein L28(RPL28) |
| RNPS1 | RNA binding protein with serine rich domain 1(RNPS1) | R3HDM1 | R3H domain containing 1(R3HDM1) | uba7 | ubiquitin like modifier activating enzyme 7(UBA7) | PLD2 | phospholipase D2(PLD2) |
| CARD8 | caspase recruitment domain family member 8(CARD8) | PAK2 | p21 (RAC1) activated kinase 2(PAK2) | MCM4 | minichromosome maintenance complex component 4(MCM4) | CDK6 | cyclin dependent kinase 6(CDK6) |
| TNFRSF1B | TNF receptor superfamily member 1B(TNFRSF1B) | STAM2 | signal transducing adaptor molecule 2(STAM2) | FYN | FYN proto-oncogene, Src family tyrosine kinase(FYN) | KIAA0391 | KIAA0391(KIAA0391) |
| TNFRSF1A | TNF receptor superfamily member 1A(TNFRSF1A) | PAK3 | p21 (RAC1) activated kinase 3(PAK3) | MCM5 | minichromosome maintenance complex component 5(MCM5) | SSR1 | signal sequence receptor subunit 1(SSR1) |
| CEBPG | CCAAT/enhancer binding protein gamma(CEBPG) | PPP2R5E | protein phosphatase 2 regulatory subunit B'epsilon(PPP2R5E) | PCSK5 | proprotein convertase subtilisin/kexin type 5(PCSK5) | NKAP | NFKB activating protein(NKAP) |
| RAD51 | RAD51 recombinase(RAD51) | LSM7 | LSM7 homolog, U6 small nuclear RNA and mRNA degradation associated(LSM7) | RUNX1 | runt related transcription factor 1(RUNX1) | GMCL1 | germ cell-less, spermatogenesis associated 1(GMCL1) |
| PAPOLA | poly(A) polymerase alpha(PAPOLA) | VIM | vimentin(VIM) | CKAP2 | cytoskeleton associated protein 2(CKAP2) | arhgef1 | Rho guanine nucleotide exchange factor 1(ARHGEF1) |
| BAK1 | BCL2 antagonist/killer 1(BAK1) | PPP3R1 | protein phosphatase 3 regulatory subunit B, alpha(PPP3R1) | NFKBID | NFKB inhibitor delta(NFKBID) | FAM58A | family with sequence similarity 58 member A(FAM58A) |
| MSH2 | mutS homolog 2(MSH2) | IL16 | interleukin 16(IL16) | CASP6 | caspase 6(CASP6) | PTPN11 | protein tyrosine phosphatase, non-receptor type 11(PTPN11) |
| MRPL19 | mitochondrial ribosomal protein L19(MRPL19) | PICK1 | protein interacting with PRKCA 1(PICK1) | FBRS | fibrosin(FBRS) | CCNE2 | cyclin E2(CCNE2) |
| SEC24B | SEC24 homolog B, COPII coat complex component(SEC24B) | EZH2 | enhancer of zeste 2 polycomb repressive complex 2 subunit(EZH2) | CASP3 | caspase 3(CASP3) | TRMT2A | tRNA methyltransferase 2 homolog A(TRMT2A) |
| TNXB | tenascin XB(TNXB) | STK19 | serine/threonine kinase 19(STK19) | CCL2 | C-C motif chemokine ligand 2(CCL2) | PTPN6 | protein tyrosine phosphatase, non-receptor type 6(PTPN6) |
| BARD1 | BRCA1 associated RING domain 1(BARD1) | PPP1R8 | protein phosphatase 1 regulatory subunit 8(PPP1R8) | PSMB1 | proteasome subunit beta 1(PSMB1) | GSK3B | glycogen synthase kinase 3 beta(GSK3B) |
| HDAC7 | histone deacetylase 7(HDAC7) | IL12RB2 | interleukin 12 receptor subunit beta 2(IL12RB2) | Cox4I1 | cytochrome c oxidase subunit 4I1(COX4I1) | SMC3 | structural maintenance of chromosomes 3(SMC3) |
| BAX | BCL2 associated X, apoptosis regulator(BAX) | ABI3 | ABI family member 3(ABI3) | GRAMD4 | GRAM domain containing 4(GRAMD4) | CDC25C | cell division cycle 25C(CDC25C) |
| KIAA0368 | KIAA0368(KIAA0368) | PARP3 | poly(ADP-ribose) polymerase family member 3(PARP3) | CASP7 | caspase 7(CASP7) | PDLIM1 | PDZ and LIM domain 1(PDLIM1) |
| SMARCC1 | SWI/SNF related, matrix associated, actin dependent regulator of chromatin subfamily c member 1(SMARCC1) | PSMB10 | proteasome subunit beta 10(PSMB10) | WBP2 | WW domain binding protein 2(WBP2) | PTPN18 | protein tyrosine phosphatase, non-receptor type 18(PTPN18) |
| CENPF | centromere protein F(CENPF) | EDN1 | endothelin 1(EDN1) | MAP3K7 | mitogen-activated protein kinase kinase kinase 7(MAP3K7) | PTPRB | protein tyrosine phosphatase, receptor type B(PTPRB) |
| NLRP1 | NLR family pyrin domain containing 1(NLRP1) | IKBKG | inhibitor of kappa light polypeptide gene enhancer in B-cells, kinase gamma(IKBKG) | EP300 | E1A binding protein p300(EP300) | CDC42 | cell division cycle 42(CDC42) |
| COG4 | component of oligomeric golgi complex 4(COG4) | ESCO2 | establishment of sister chromatid cohesion N-acetyltransferase 2(ESCO2) | LDHB | lactate dehydrogenase B(LDHB) | PSME3 | proteasome activator subunit 3(PSME3) |
| TOP2A | topoisomerase (DNA) II alpha(TOP2A) | NEIL3 | nei like DNA glycosylase 3(NEIL3) | NEK2 | NIMA related kinase 2(NEK2) | USP6NL | USP6 N-terminal like(USP6NL) |
| BCL6 | B-cell CLL/lymphoma 6(BCL6) | SLC7A7 | solute carrier family 7 member 7(SLC7A7) | PXN | paxillin(PXN) | GRB2 | growth factor receptor bound protein 2(GRB2) |
| TP53BP1 | tumor protein p53 binding protein 1(TP53BP1) | PIAS2 | protein inhibitor of activated STAT 2(PIAS2) | ARHGAP12 | Rho GTPase activating protein 12(ARHGAP12) | DCLRE1C | DNA cross-link repair 1C(DCLRE1C) |
| PLSCR3 | phospholipid scramblase 3(PLSCR3) | ABCB1 | ATP binding cassette subfamily B member 1(ABCB1) | AURKB | aurora kinase B(AURKB) | TRAF2 | TNF receptor associated factor 2(TRAF2) |
| PARP1 | poly(ADP-ribose) polymerase 1(PARP1) | USF2 | upstream transcription factor 2, c-fos interacting(USF2) | PDCL3 | phosducin like 3(PDCL3) | RFXANK | regulatory factor X associated ankyrin containing protein(RFXANK) |
| YES1 | YES proto-oncogene 1, Src family tyrosine kinase(YES1) | MED16 | mediator complex subunit 16(MED16) | CTNNB1 | catenin beta 1(CTNNB1) | kras | KRAS proto-oncogene, GTPase(KRAS) |
| ERCC4 | ERCC excision repair 4, endonuclease catalytic subunit(ERCC4) | PTCH1 | patched 1(PTCH1) | RINT1 | RAD50 interactor 1(RINT1) | HUS1 | HUS1 checkpoint clamp component(HUS1) |
| ADH5 | alcohol dehydrogenase 5 (class III), chi polypeptide(ADH5) | CXXC1 | CXXC finger protein 1(CXXC1) | RCAN3 | RCAN family member 3(RCAN3) | IPO5 | importin 5(IPO5) |
| ERCC6 | ERCC excision repair 6, chromatin remodeling factor(ERCC6) | IBTK | inhibitor of Bruton tyrosine kinase(IBTK) | prkd2 | protein kinase D2(PRKD2) | ESCO1 | establishment of sister chromatid cohesion N-acetyltransferase 1(ESCO1) |
| XRCC5 | X-ray repair cross complementing 5(XRCC5) | MTOR | mechanistic target of rapamycin(MTOR) | N4BP2 | NEDD4 binding protein 2(N4BP2) | PCTP | phosphatidylcholine transfer protein(PCTP) |
| G3BP2 | G3BP stress granule assembly factor 2(G3BP2) | dok2 | docking protein 2(DOK2) | SOCS1 | suppressor of cytokine signaling 1(SOCS1) | FAM8A1 | family with sequence similarity 8 member A1(FAM8A1) |
| STAM | signal transducing adaptor molecule(STAM) | PTGES3 | prostaglandin E synthase 3(PTGES3) | ENO1 | enolase 1(ENO1) | ICAM1 | intercellular adhesion molecule 1(ICAM1) |
